# Development and Evaluation of a Dietary Polysaccharide-Stabilized Peptide-Fe^2+^ Complex Beverage for Iron Deficiency Anemia Intervention

**DOI:** 10.3390/foods15203644

**Published:** 2026-10-14

**Authors:** Yongqian Liang, Shanting Lin, Shengjun Chen, Yongqiang Zhao, Hui Huang, Zhenxing Li, Xiao Hu

**Affiliations:** 1 College of Pharmacy, Guangdong Pharmaceutical University, Guangzhou 510006, China; liangyongqian@gdpu.edu.cn; 2 Key Laboratory of Aquatic Product Processing, Ministry of Agriculture and Rural Affairs, South China Sea Fisheries Research Institute, Chinese Academy of Fishery Sciences, Guangzhou 510300, China; chenshengjun@scsfri.ac.cn (S.C.); zhaoyq@scsfri.ac.cn (Y.Z.); huanghuigd@aliyun.com (H.H.); 3 State Key Laboratory of Marine Food Processing & Safety Control, College of Food Science and Engineering, Ocean University of China, Qingdao 266400, China; lizhenxing@ouc.edu.cn; 4 Institute of Agro-Products Processing Science and Technology, Guangxi Academy of Agricultural Sciences, Nanning 530007, China; 5 Sanya Tropical Fisheries Research Institute, Sanya 572000, China

**Keywords:** iron deficiency anemia, peptide iron complex, dietary polysaccharide stabilization, composite beverage optimization, sensory and suspension stability

## Abstract

This study developed a dietary polysaccharide-stabilized peptide-Fe^2+^ composite beverage to address iron deficiency anemia (IDA). Through single-factor and orthogonal experiments, the formulation was optimized (cranberry/blueberry concentrate ratio 4:6, 600 mL/L; FA-COS@Peptide-Fe^2+^@PTry-CMC 200 mL/L; vitamin C 1.5 g/L; pectin 0.06 g/L), and processing parameters were determined (homogenization: 12,000 rpm, 20 min; sterilization: 95 °C, 15 min). Storage stability was optimal at 4 °C in dark conditions. Under the optimal processing conditions, the beverage attained a maximum suspension stability of 17.74% and the optimized formulation achieved a highest sensory score of 94.0 (out of 100). In vivo experiments showed that the beverage dose-dependently restored hematological parameters (e.g., HGB, SI, TIBC) and tissue iron levels, alleviated intestinal oxidative stress and inflammatory markers in IDA mice, while repairing colonic mucosal damage without adverse effects. The beverage demonstrated high iron-supplementing efficacy and excellent biosafety, offering a promising strategy for IDA intervention.

## 1. Introduction

Iron is an essential trace element that serves as a central component in hemoglobin (HGB), myoglobin, and key enzymes involved in oxygen transport, energy metabolism, DNA synthesis, and immune function [1,2]. However, iron deficiency remains one of the most prevalent global nutritional disorders, affecting billions worldwide, especially children, women of childbearing age, and pregnant women, leading to iron deficiency anemia (IDA) [3,4,5]. IDA contributes to fatigue, impaired cognition, weakened immunity, and growth delays, imposing substantial health and socioeconomic burdens [6,7,8].

Current iron supplementation strategies, including oral inorganic salts (e.g., ferrous sulfate) and iron-fortified foods, present significant limitations. Inorganic salts frequently cause gastrointestinal distress, while their absorption is inhibited by dietary compounds such as phytates and tannins, resulting in low bioavailability [9,10]. Although natural iron-rich foods are available, their iron content is often insufficient and variable, complicating reliable and rapid nutritional intervention. Peptide-Fe^2+^ complexes have therefore gained attention as promising alternatives. Formed by chelating Fe^2+^ with peptides from enzymatically hydrolyzed proteins, these complexes enhance iron absorption via peptide-specific pathways and reduce gastrointestinal side effects [11,12,13]. While our prior studies [11,12,13] focused on the synthesis, basic characterization, and in vitro iron-chelating properties of peptide-Fe^2+^ complexes, they did not address the critical challenges of stability, palatability, and practical delivery in a beverage format.

To overcome these issues, this study introduced a stabilization approach using a polysaccharide-based delivery system. By encapsulating peptide-Fe^2+^ complexes within folate-chitosan oligosaccharide (FA-COS) and phosphotyrosine-carboxymethyl cellulose (PTry-CMC) nanoparticles (FA-COS@Peptide-Fe^2+^@PTry-CMC), the formulation prevented oxidation and improved dispersion stability, thereby maintaining efficacy during storage. A beverage format further enhanced practicality. Its liquid nature facilitates ingestion for pediatric and geriatric populations, while flavoring agents such as cranberry–blueberry blends mask metallic tastes. This convenient, palatable form supported daily consumption and improved adherence, particularly among selective eaters. Overall, this approach not only effectively prevented oxidation and precipitation of the iron complex during storage but also enabled its seamless integration into a palatable, ready to drink beverage. Furthermore, this work was the first to systematically optimize the beverage formulation (including cranberry/blueberry concentrates, vitamin C, and pectin), processing parameters (homogenization speed/time, sterilization), evaluated sensory and suspension stability, and demonstrated dose-dependent efficacy and safety in an in vivo IDA mouse model. Thus, the current manuscript represents a significant extension beyond our previous work, moving from basic complex synthesis to a fully formulated, stable, and efficacious iron-fortified beverage for IDA intervention.

## 2. Materials and Methods

### 2.1. Materials

Cranberry and blueberry concentrates were procured from Northeast Specialty Yuanweizhenpin. Food-grade citric acid, vitamin C, pectin, xanthan gum, sodium carboxymethyl cellulose (CMC-Na), and FeSO_4_ were supplied by Zhongchen Biotechnology Co., Ltd. Zhengzhou, China. Microbial total plate count test sheets were sourced from Guangdong Huankai Microbial Technology Co., Ltd. Gaungzhou, China. AIN-93G standard diet (45 mg/kg iron) and low-iron diet (5 mg/kg iron) were provided by Anhui Kuibu Shuyu Biotechnology Co., Ltd. Xuancheng, China. The HGB assay kit was purchased from Nanjing Jiancheng Bioengineering Institute Nanjing, China. Assay kits for total antioxidant capacity (T-AOC), catalase (CAT), superoxide dismutase (SOD), glutathione peroxidase (GSH-Px/GPX), malondialdehyde (MDA), serum iron (SI), and total iron-binding capacity (TIBC) were acquired from Beijing Solarbio Science & Technology Co., Ltd. Beijing, China. Mouse ELISA kits for tumor necrosis factor-α (TNF-α), interleukin-6 (IL-6), and interleukin-10 (IL-10) were obtained from Chengdu Rinas Technology Co., Ltd. Chengdu, China.

### 2.2. Preparation of FA-COS@Peptide-Fe^2+^@PTry-CMC

Peptide-Fe^2+^ were prepared following a previously reported protocol by our group [11,13]. First, collagen was extracted from tilapia skin and enzymatically hydrolyzed using trypsin at an enzyme-to-substrate ratio of 2%, pH 7.5, 37 °C, for 2 h to yield Fe^2+^-chelating peptides. The peptide-Fe^2+^ complex was then synthesized by mixing the peptides with FeSO_4_ at a mass ratio of 20:1 (peptides: Fe^2+^) under conditions of pH 5, 25 °C, for 90 min. Subsequently, a 5 mg/mL solution of the peptide-Fe^2+^ complexes were added dropwise into 1 mg/mL FA-COS solution and vortex-mixed for 10 min. The resulting mixture was then added dropwise into a 5 mg/mL solution of PTry-CMC, followed by vortex mixing for 30 min to form the FA-COS@Peptide-Fe^2+^@PTry-CMC nanoparticles.

### 2.3. Optimization of Raw Material Ratios in the Composite Beverage

Single-factor experiments were performed to assess the effects of the following variables on the sensory properties of the beverage: (i) the volumetric ratio of cranberry to blueberry concentrate and its total addition level, (ii) the dosage of FA-COS@Peptide-Fe^2+^@PTry-CMC, and (iii) the vitamin C concentration. The base formulation consisted of FA-COS@Peptide-Fe^2+^@PTry-CMC (100 mL/L), vitamin C (0.5 g/L), and pectin (0.2 g/L), followed by homogenization at 10,000 rpm for 10 min and sterilization at 100 °C for 10 min.

(1) Using the base formulation, the cranberry-to-blueberry concentrate ratio (8:2, 7:3, 6:4, 5:5, 4:6, 3:7, and 2:8, *v*/*v*) and total concentrate addition level (200–700 mL/L) were evaluated for their impact on sensory attributes.
(2) With the optimal concentrate ratio and addition level from step (1), the effect of FA-COS@Peptide-Fe^2+^@PTry-CMC concentration (50–300 mL/L) on sensory properties was tested.
(3) Based on the parameters established in steps (1) and (2), the influence of vitamin C concentration (0–2.5 g/L) on beverage sensory quality was examined.

Sensory assessment was conducted by a panel of 10 trained evaluators (gender-balanced) with documented experience in descriptive analysis. Panelists evaluated color, texture, aroma, and taste. Between samples, they rinsed with water and observed a 1 min interval to minimize carryover effects. Detailed scoring criteria are provided in Table 1.

### 2.4. Orthogonal Design for Optimizing the Beverage Base Formula

Building upon the single-factor experiments, an orthogonal array design was implemented to optimize the beverage base formulation. The factors and levels selected were as follows: cranberry/blueberry concentrate blend (4:6, *v*/*v*) at 500, 600, and 700 mL/L; FA-COS@Peptide-Fe^2+^@PTry-CMC at 150, 200, and 250 mL/L; and vitamin C at 1.0, 1.5, and 2.0 g/L. The experimental runs and corresponding parameters are summarized in Table 2, with sensory evaluation serving as the primary response.

### 2.5. Optimization of Stabilizers and Stabilization Parameters in the Composite Beverage

Single-factor experiments were conducted to optimize the type and concentration of stabilizers and the homogenization conditions for the composite beverage, using the base formulation established in Section 2.4. The initial homogenization condition was set at 8000 rpm for 10 min.

(1) The influence of different stabilizers, carboxymethyl cellulose sodium (CMC-Na), xanthan gum, and pectin at concentrations ranging from 0 to 0.1 g/L on suspension stability was examined.
(2) Using the optimal stabilizer and concentration from step (1), the impact of homogenization speed (2000–12,000 rpm, for 10 min) on suspension stability was evaluated.
(3) Based on the outcomes of steps (1) and (2), the effect of homogenization time (3–25 min) on suspension stability was tested.

Suspension stability (SS, %) was determined according to the previous method. A 3 mL aliquot of the beverage was centrifuged at 4000 r/min for 15 min. After standing for 10 min, the transmittance (T, %) of the supernatant was measured at 625 nm against a distilled water blank using a UV spectrophotometer. SS was calculated as:

$$\mathrm{SS}\left(\%\right)=\frac{T0}{T1}\times 100\%$$

where T0 and T1 represented the transmittance (%) of the sample before and after centrifugation, respectively. SS is a centrifugal stability index calculated as T_0_/T_1_ × 100%; lower values indicate greater centrifugal precipitation.

### 2.6. Orthogonal Experiment on the Effects of Stabilizers and Stabilization Parameters on the Suspension Stability of the Composite Beverage

Building upon the single-factor experiments, an orthogonal array design was implemented to optimize the stabilization conditions for the composite beverage. The selected factors and levels were pectin concentration (0.04, 0.06, 0.08 g/L), homogenization speed (8000, 10,000, 12,000 rpm), and homogenization time (15, 20, 25 min). The experimental layout and corresponding parameters are detailed in Table 3. Suspension stability served as the response variable.

### 2.7. Effect of Different Sterilization Conditions on Microbial Indexes of the Composite Beverage

The prepared composite beverage, based on the optimized formulation and processing parameters from Section 2.4 and Section 2.6, was used to evaluate the effects of sterilization conditions on microbial quality. Sterilization was performed at temperatures of 60, 70, 80, 90, and 100 °C, each combined with holding times of 10, 15, 20, 25, and 30 min. The total plate count (TPC) was measured following the manufacturer’s instructions (Guangdong Huankai Microbial Technology Co., Ltd. Guanzghou, China) to assess microbial load.

### 2.8. Effects of Light and Temperature on Beverage Stability During Storage

To evaluate storage stability, beverages were stored under five conditions: 4 °C (dark), 25 °C (dark), 40 °C (dark), 4 °C (normal light), and 25 °C (normal light). Samples were collected on days 1, 3, 5, 7, and 9 for analysis. Absorbance was measured at 420, 520, and 620 nm using a UV–Vis spectrophotometer (Lambda 365, PerkinElmer, Waltham, MA, USA) according to a published method. Color intensity (I) was calculated as I = A_420_ + A_520_ + A_620_, and hue (N) as N = A_420_/A_520_. The pH of each sample was also measured. To determine suspension stability, samples were centrifuged at 4000 r/min for 10 min, and absorbance at 660 nm was recorded before (*A*1) and after (*A*2) centrifugation. The change rate was calculated as:

$$\mathrm{Rate}\;\mathrm{of}\;\mathrm{Change}\;\%\;=\;\frac{A1}{A2}\times 100$$

where *A*1 and *A*2 represent the absorbance values before and after centrifugation, respectively.

### 2.9. General Preparation Procedure of the Dietary Polysaccharide-Stabilized Peptide-Fe^2+^ Compo Site Beverage

The dietary polysaccharide-stabilized peptide-Fe^2+^ composite beverage was prepared according to the following procedure: cranberry/blueberry concentrate was added, followed by FA-COS@Peptide-Fe^2+^@PTry-CMC, vitamin C, and pectin. The mixture was then diluted with purified water to a final volume of 1 L, homogenized, filled into containers, sterilized, and finally obtained as the finished dietary polysaccharide-stabilized peptide-Fe^2+^ composite beverage. The ingredient ratios, homogenization speed/time, sterilization conditions are determined in the results (Section 3.10).

### 2.10. Iron Supplementation Effect of the Composite Beverage in IDA Mice

All animal experimental procedures were performed in strict accordance with the ARRIVE 2.0 guidelines (Animal Research: Reporting of In Vivo Experiments) and were approved by the Ethics Committee of the South China Sea Fisheries Research Institute, Chinese Academy of Fishery Sciences (Approval No.: nhdf2025-27) and conducted in compliance with EU Directive 2010/63/EU. Three-week-old male SPF-grade ICR mice were purchased from Guangdong Provincial Medical Laboratory Animal Center [SCXK (Yue) 2022-0002] and housed under controlled temperature, humidity, and light/dark cycles with free access to AIN-93G diet and deionized water during acclimation. To induce IDA, mice were randomly assigned to either a control group (standard AIN-93G diet, 45 mg Fe/kg) or IDA model group (low-iron diet, 5 mg Fe/kg) for 4 weeks. Each group was housed in a separate room, and consisted of exactly five mice (*n* = 5 per group). IDA was confirmed when tail-vein blood Hb levels, measured using a commercial kit, fell below 100 g/L.

From week 5, control mice continued on the standard diet with saline gavage. IDA mice were divided into four groups: (1) IDA control (low-iron diet + saline); (2) low-dose beverage (low-iron diet + beverage providing, 1.0 mg Fe^2+^/kg BW); (3) medium-dose (low-iron diet + beverage providing, 2.0 mg Fe^2+^/kg BW); and (4) high-dose (low-iron diet + beverage providing, 4.0 mg Fe^2+^/kg BW). Interventions lasted 21 days, with body weight recorded weekly. After overnight fasting, mice were anesthetized and blood was collected. Serum was separated by centrifugation (4000 rpm, 15 min) for analysis of serum iron (SI) and total iron-binding capacity (TIBC). Euthanasia was performed by cervical dislocation. The spleen, liver, and heart were excised, rinsed, blotted, and weighed for organ index calculation. Duodenal tissues were collected for oxidative stress and inflammatory cytokine assays. Colon tissues were fixed in 4% paraformaldehyde for histopathological examination.

### 2.11. Statistical Analysis

Statistical analysis was performed using SPSS 20.0. Data are expressed as mean ± SE. For animal studies, *n* = 5 per group; for all other experiments, *n* = 3 independent replicates. Differences between groups were assessed by one-way ANOVA with Tukey’s multiple comparisons, and a *p*-value < 0.05 was considered statistically significant. For all in vivo data, normality was assessed using the Shapiro–Wilk test and homogeneity of variance was evaluated using Levene’s test prior to ANOVA. Data meeting both assumptions (*p* > 0.05) were analyzed by one-way ANOVA followed by Tukey’s multiple comparisons test. Figures were prepared using Origin 9.

## 3. Results

### 3.1. Effects of Cranberry/Blueberry Concentrate Volume Ratio and Addition Level on the Sensory Properties of the Composite Beverage

**Figure 1:**
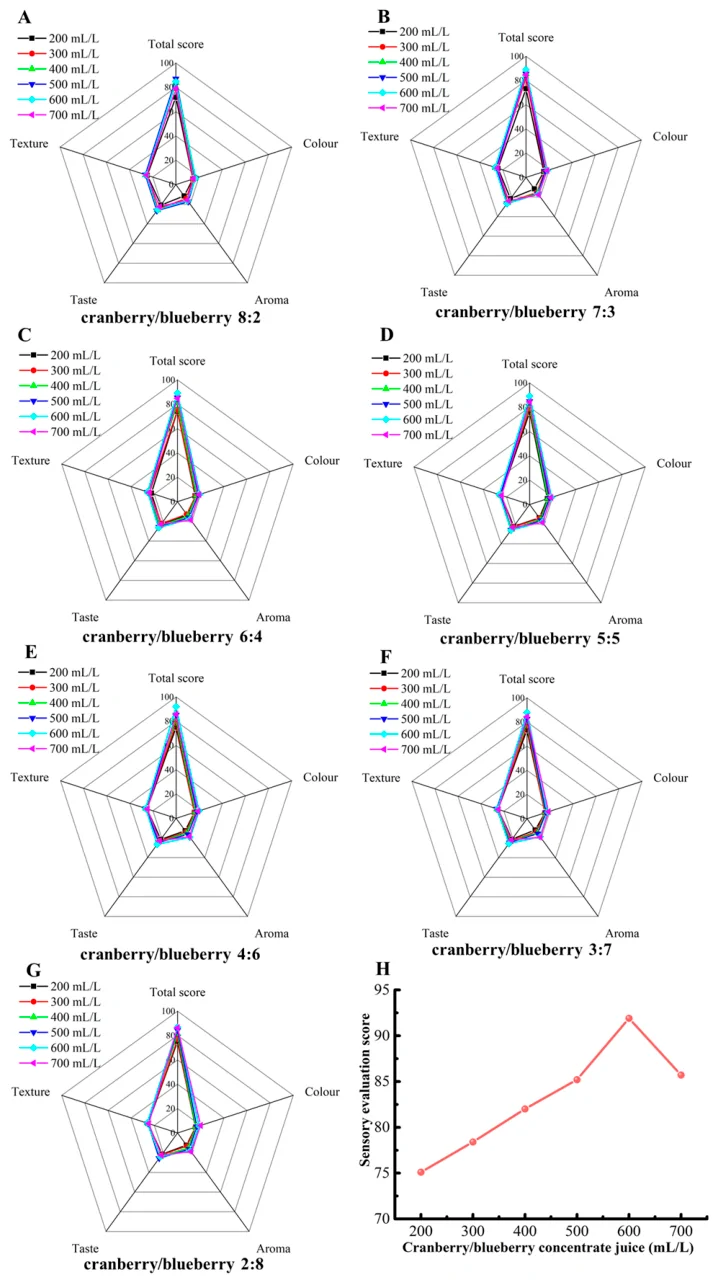
Effects of different volume ratios and addition amounts of cranberry/blueberry beverages on the sensory properties of composite beverages. (**A**–**G**) Sensory radar images under the conditions of a Cranberry/Blueberry ratio of 8:2, 7:3, 6:4, 5:5, 4:6, 3:7, and 2:8, respectively. (**H**) Sensory evaluation score of different cranberry/blueberry juice addition levels.

The volume ratio of cranberry/blueberry concentrate significantly influenced the sensory profile of the beverage. Radar charts (Figure 1A–G) demonstrated distinct sensory patterns across ratios ranging from 8:2 to 2:8. Higher cranberry proportions (e.g., 8:2, 7:3) enhanced color and odor but reduced taste scores, likely due to elevated acidity. Increasing the blueberry ratio (e.g., 5:5, 4:6) improved taste balance and overall acceptability, with the most balanced sensory attributes observed at 5:5 and 4:6. Next, the effect of addition level was evaluated. As illustrated in Figure 1H, sensory scores of the composite beverage first increased and then decreased as the addition level rose from 200 to 700 mL/L. The highest overall scores were achieved at addition levels of 500–600 mL/L, suggesting this range as the optimal concentration. Excessive addition (e.g., 700 mL/L) may lead to overpowering flavor or textural imbalance, resulting in lower scores. Interaction between ratio and concentration was evident in blueberry-dominant blends (e.g., 3:7, 2:8), higher concentrations improved odor and taste more than color, whereas in cranberry-rich blends, color and sourness were more concentration-sensitive. Overall, optimal sensory quality was achieved at a cranberry/blueberry ratio between 5:5 and 4:6 and a total concentrate level of 500–600 mL/L, balancing color, odor, taste, and texture for maximal acceptability.

### 3.2. Effect of FA-COS@Peptide-Fe^2+^@PTry-CMC Addition Level on the Sensory Properties of the Composite Beverage

**Figure 2:**
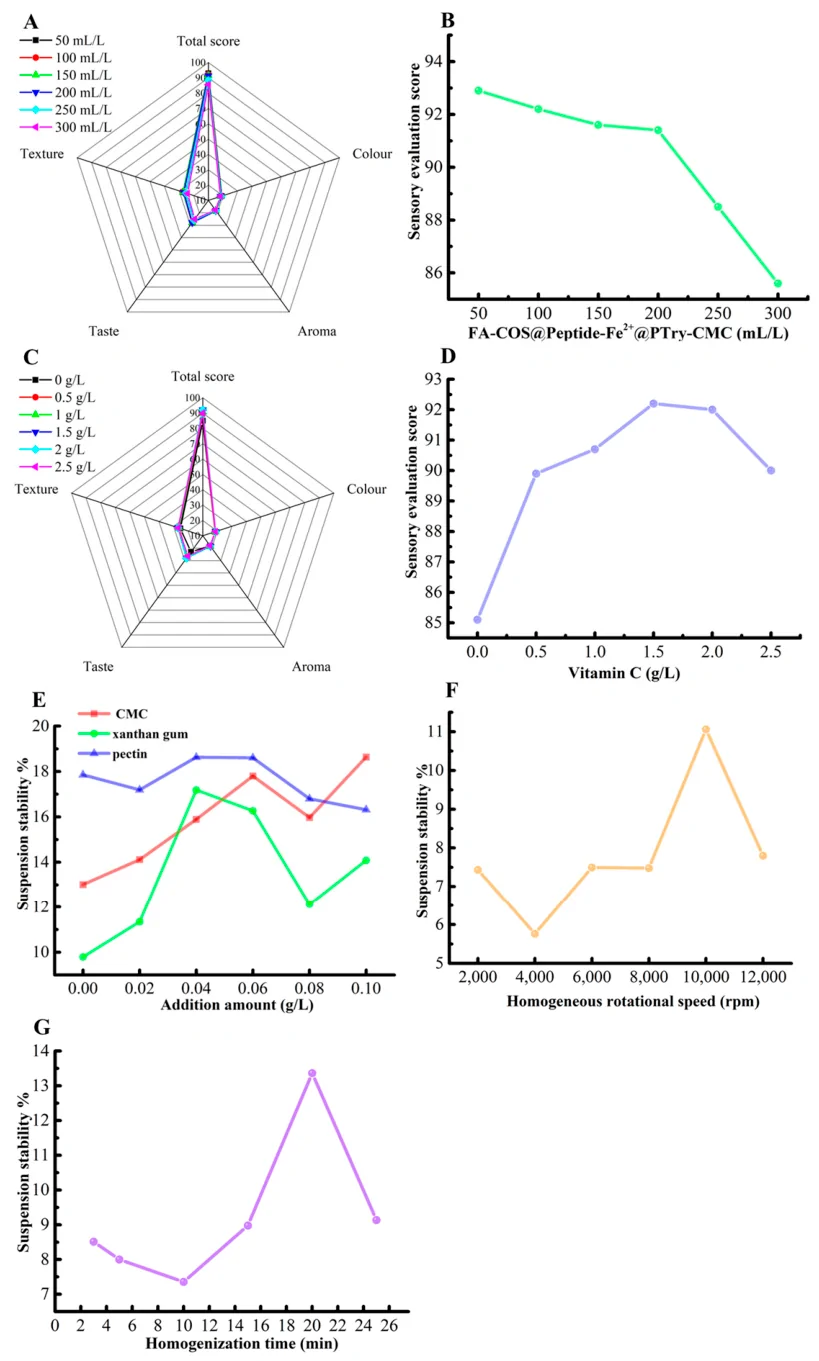
Effects of formulation and processing parameters on the sensory properties and suspension stability of the composite beverage. (**A**) Sensory radar images of different FA-COS@Peptide-Fe^2+^@PTry-CMC addition. (**B**) Sensory evaluation score of different FA-COS@Peptide-Fe^2+^@PTry-CMC addition. (**C**) Sensory radar images of different vitamin C addition. (**D**) Sensory evaluation score of different vitamin C addition. (**E**) Suspension stability of different stabilizer addition. (**F**) Suspension stability of different homogenization speed. (**G**) Suspension stability of different homogenization time.

Varying the addition level of FA-COS@Peptide-Fe^2+^@PTry-CMC affected multiple sensory dimensions, including color, odor, taste, and texture (Figure 2A). At 200 mL/L, the radar chart showed the most balanced profile, indicating optimal performance across all attributes. Below and above this level, deficits emerged in specific dimensions, such as taste harmony and texture. Overall sensory scores remained high and stable from 50 to 200 mL/L but decreased steadily beyond 200 mL/L (Figure 2B). This suggested that within 50–200 mL/L, the nanoparticles were well-compatible with the beverage system and did not compromise sensory quality. However, above 200 mL/L, overall acceptability declined significantly, reflecting a sensory acceptance threshold beyond which negative effects (e.g., impaired taste) became dominant. Thus, 200 mL/L was identified as the optimal addition level, providing the best balance of color, odor, taste, and texture.

### 3.3. Effect of Vitamin C Addition Level on the Sensory Properties of the Composite Beverage

Vitamin C addition level significantly affected the sensory profile of the beverage (Figure 2C). The radar chart showed the most balanced profile at 1.5 g/L, whereas deviations from this level resulted in deficits in specific sensory attributes. Overall sensory scores followed an inverted U-shaped trend with increasing vitamin C addition level (0–2.5 g/L), peaking at 1.5 g/L (Figure 2D). Lower concentrations (0–0.5 g/L) yielded reduced scores, likely due to insufficient antioxidant and flavor-enhancing effects. At higher levels (2.0–2.5 g/L), taste and odor scores declined markedly, possibly because of excessive acidity or textural changes, although color remained largely unaffected. Thus, 1.5 g/L was identified as the optimal vitamin C addition level, providing the best balance among color, odor, taste, and texture and yielding the highest overall sensory score.

### 3.4. Orthogonal Experiment for Optimizing the Base Formulation of the Composite Beverage

An L_9_(3^4^) orthogonal design (Table 4) was employed to optimize the beverage formulation with three factors, cranberry/blueberry concentrate (A), FA-COS@Peptide-Fe^2+^@PTry-CMC (B), and vitamin C (C), each at three levels. Sensory scores ranged from 88.7 to 94.0, with formulation A_2_B_2_C_2_ (run no. 5) achieving the highest score (94.0). Range analysis indicated that factor A had the greatest influence on sensory scores (R = 3.00), followed by factor C (R = 2.60), and then factor B (R = 0.90). The small range for the blank column (R = 0.70) suggested low experimental error. K-value analysis identified the optimal level for each factor as A_2_ (K_2_ = 92.77), B_2_ (K_2_ = 91.87), and C_2_ (K_2_ = 92.37). Thus, the optimum combination derived from range analysis was A_2_B_2_C_2_, which corresponded to the highest-scoring experimental run. For factor A, the K values (K_1_ = 91.47, K_2_ = 92.77, K_3_ = 89.77) indicated that moderate addition enhanced sensory quality, whereas excessive addition reduced it. Similarly, both factors B and C exhibited peak scores at their middle levels, demonstrating a clear optimum concentration. In summary, orthogonal optimization determined the following optimal formulation: cranberry/blueberry concentrate (level 2), FA-COS@Peptide-Fe^2+^@PTry-CMC (level 2), and vitamin C (level 2), which collectively yielded the highest sensory score.

### 3.5. Effect of Different Stabilizer Addition Level on the Suspension Stability of the Composite Beverage

Suspension stability increased significantly with pectin concentration up to 0.04 g/L, rising from 10% to 18%, and remained at this peak level between 0.04 and 0.06 g/L (Figure 2E). Compared with CMC and xanthan gum, pectin achieved the highest stability (18%) at a lower concentration (0.04 g/L), while CMC required 0.10 g/L to reach 16% and xanthan gum only 14% at the same concentration, indicating superior stabilizing efficiency of pectin. As a hydrophilic polysaccharide, pectin likely forms a three-dimensional network that increased viscosity and provides steric hindrance, effectively inhibiting particle sedimentation [14]. The plateau in stability above 0.06 g/L may reflect excessive chain entanglement leading to heterogeneous viscosity, consistent with the typical plateau effect of polysaccharide stabilizers. Thus, the optimal pectin concentration for maximizing suspension stability was 0.04–0.06 g/L, at which pectin outperformed CMC and xanthan gum in both effectiveness and efficiency.

### 3.6. Effect of Homogenization Speed on the Suspension Stability of the Composite Beverage

The effect of homogenization speed on beverage suspension stability is shown in Figure 2F. Stability initially decreased from 7.0% to 5.8% at low speeds (2000–4000 rpm), possibly due to insufficient dispersion. It then recovered to 7.0% at 6000 rpm, dipped briefly at 8000 rpm (6.0%), and peaked at 10,000 rpm (11.0%). A further increase to 12,000 rpm reduced stability to 7.8%, suggesting particle breakage or flocculation at excessive shear. Thus, 10,000 rpm was optimal, providing adequate energy for dispersion without destabilizing the system. This indicated a clear processing window, with both lower (e.g., 4000 rpm) and higher (12,000 rpm) speeds yielding lower stability. Increasing speed moderately (6000–10,000 rpm) improved shear-induced distribution of stabilizers (e.g., pectin) and promoted network formation, enhancing stability. Conversely, overly high speeds may degrade stabilizer chains, increase flocculation, or cause local overheating [15]. Therefore, homogenization between 8000 and 10,000 rpm balanced sufficient dispersion energy with minimal structural damage, achieving maximum suspension stability (11.0%) at 10,000 rpm.

### 3.7. Effect of Homogenization Time on the Suspension Stability of the Composite Beverage

Suspension stability was strongly influenced by homogenization time (Figure 2G). Maximum stability (13.5%) was achieved at 20 min, significantly higher than at shorter or longer times (*p* < 0.05), indicating that 20 min of homogenization optimally activated the stabilizers. Low stability during the initial phase (3–10 min) likely resulted from insufficient time for complete particle dispersion and stabilizer network development. A plateau between 10 and 15 min suggested a balance between particle refinement and stabilizer hydration. The sharp increase from 15 to 20 min corresponded to full hydration and network formation of stabilizers such as pectin, leading to optimal particle dispersion, surface saturation, and maximal steric hindrance. Beyond 20 min, stability declined rapidly, possibly due to shear-induced degradation of stabilizer chains and localized heating, which might disrupt system stability [16]. Thus, 20 min was the recommended homogenization time to maximize suspension stability while minimizing energy use and potential adverse effects.

### 3.8. Orthogonal Experiment on the Effects of Different Factors on the Suspension Stability of the Composite Beverage

As shown in Table 5, range analysis indicated that homogenization speed (R = 7.333) had the greatest influence on suspension stability, followed by pectin concentration (R = 3.446) and homogenization time (R = 1.530). The small error term (R = 0.897) confirmed the reliability of the design. Level-effect analysis showed that a moderate pectin concentration (level 2, K_2_ = 14.973) was optimal. High homogenization speed (level 3, K_3_ = 16.230) yielded the best stability, while a moderate homogenization time (level 2, K_2_ = 13.627) was slightly superior to shorter or longer durations. The optimal combination determined from K-values was pectin level 2, homogenization speed level 3, and time level 2. This combination (run 6) achieved the highest suspension stability (17.74%), validating its effectiveness. Homogenization speed, as the most influential factor, ensured sufficient shear for particle dispersion at high speed (level 3). Pectin at level 2 likely provided the critical concentration for gel network formation, while homogenization time at level 2 balanced dispersion efficiency with energy input.

### 3.9. Effects of Different Sterilization Conditions on Microbial Indexes of the Composite Beverage

Table 6 shows that sterilization temperature and time synergistically reduced microbial counts (total plate count, TPC) in the beverage. Higher temperatures and longer times significantly improved microbial quality. Temperature was the dominant factor. For example, at a fixed 10 min, TPC decreased from 8.5 × 10^4^ CFU/mL (60 °C) to 1.2 × 10^3^ CFU/mL (70 °C), 200 CFU/mL (80 °C), and <10 CFU/mL (90 °C and 100 °C), meeting commercial sterility standards. High temperatures likely accelerated microbial death by denaturing proteins and enzymes [17]. Longer times also reduced TPC, especially at lower temperatures. At 60 °C, extending time from 5 to 25 min gradually lowered TPC from 1.2 × 10^4^ to 2.1 × 10^2^ CFU/mL, indicating cumulative thermal dose dependence. At 90 °C, TPC fell below 10 CFU/mL within 15 min, and longer times (2025 min) ensured consistent sterility. Effective sterilization was achieved at ≥90 °C for ≥15 min or ≥100 °C for ≥5 min. Considering product quality, energy efficiency, and nutrient/flavor retention, 90 °C for 15–20 min was recommended as an optimized sterilization condition for this beverage.

### 3.10. Final Optimized Preparation Procedure of the Composite Beverage

Based on the results from the single-factor and orthogonal experiments described above, the formulation parameters and preparation process for the dietary polysaccharide-stabilized peptide-Fe^2+^ composite beverage were determined as follows: cranberry/blueberry concentrate (volume ratio 4:6), addition amount 600 mL/L → FA-COS@Peptide-Fe^2+^@PTry-CMC addition amount 200 mL/L → vitamin C addition amount 1.5 g/L → pectin addition amount 0.06 g/L → dilute with purified water to a final volume of 1 L → homogenize at 12,000 rpm for 20 min → fill into containers → sterilize at 95 °C for 15 min → obtain the final dietary polysaccharide-stabilized peptide-Fe^2+^ composite beverage.

### 3.11. Effect of the Beverage on Hematological Parameters in IDA Mice

**Figure 3:**
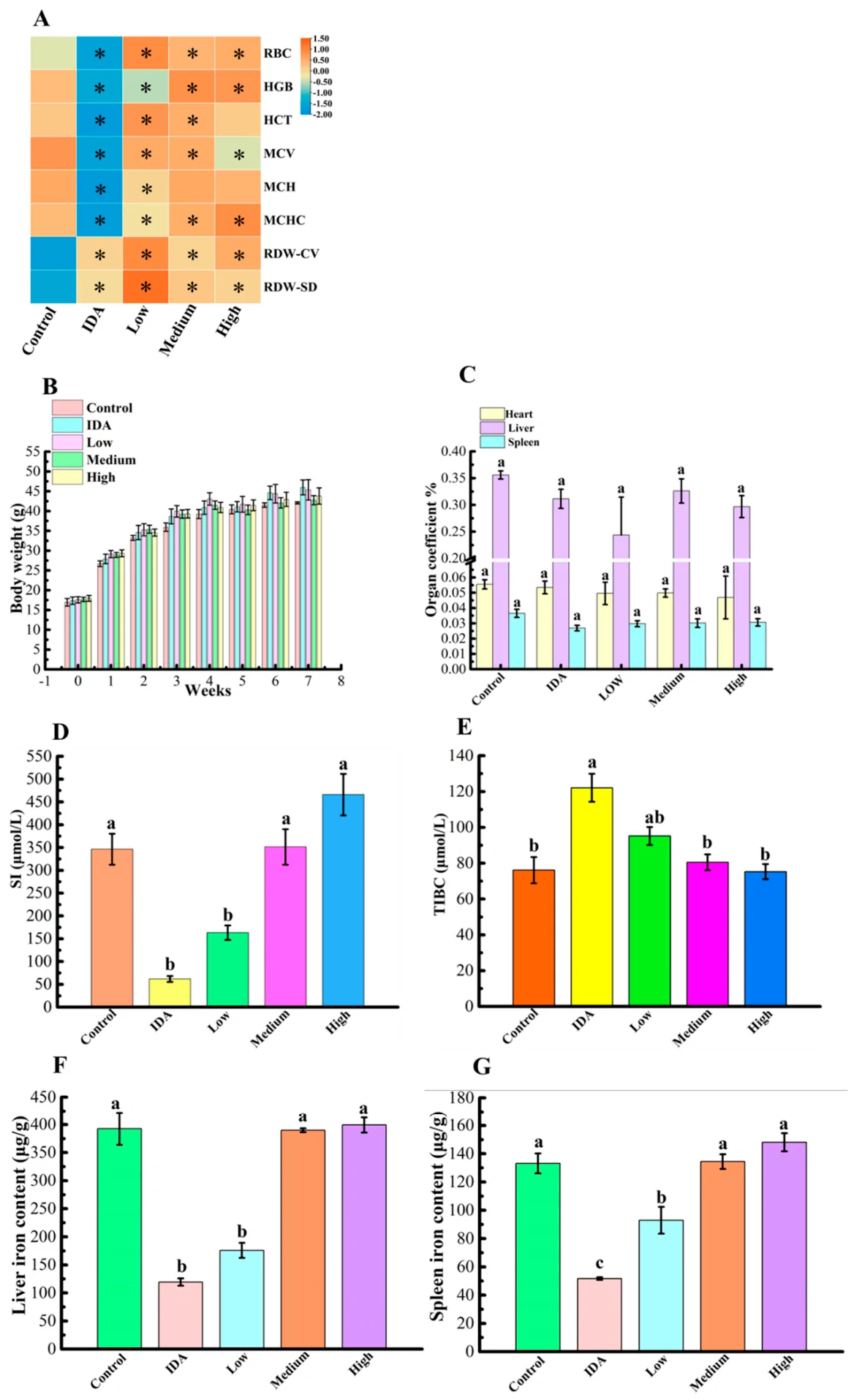
Effects of the composite beverage on hematological parameters (**A**), body weight (**B**), organ indices (**C**), serum iron indices (**D**,**E**), and tissue iron content (**F**,**G**) in IDA mice. The symbol “*” represents a significant difference compared to the control group. Different superscript letters above bars indicate significant differences between groups. The color scale indicates Z scores (standardized values) calculated relative to the mean and standard deviation of the control group, with the scale ranging from −2.00 to 1.50.

Hematological parameters were assessed in five groups: control (Control), IDA model, and low, medium, and high-dose beverage intervention groups. The beverage dose-dependently reversed IDA-induced hematological abnormalities. Compared with controls, the IDA group displayed typical microcytic hypochromic anemia. HGB, RBC, hematocrit (HCT), mean corpuscular volume (MCV), and mean corpuscular hemoglobin (MCH) were significantly reduced, while red cell distribution width—coefficient of variation (RDW-CV) and red cell distribution width—standard deviation (RDW-SD) increased, indicating anemia and anisocytosis (Figure 3A). Beverage intervention restored hematological parameters dose-dependently. Even the low dose improved HGB and RBC counts; medium and high doses restored HGB, RBC, and HCT to near-control levels and significantly increased MCV and MCH, indicating normalized red cell morphology. RDW-CV and RDW-SD also decreased, reflecting improved erythrocyte uniformity (Figure 3A). Thus, the beverage effectively alleviated iron-deficiency anemia by improving both erythrocyte quantity and quality.

### 3.12. Effect of the Composite Beverage on Body Weight, Organ Indices, Serum Iron, and Total Iron-Binding Capacity in IDA Mice

As shown in Figure 3B, the body weight of all experimental groups increased steadily over time, with essentially overlapping growth curves and no significant intergroup differences. This indicated that neither the induction of IDA nor the subsequent intervention with different beverage have any marked adverse effect on normal growth and development. Figure 3C shows that there were no significant differences in heart index or liver index among the groups, suggesting that IDA and beverage intervention did not specifically affect the relative mass of these two important metabolic organs. In contrast, spleen index was elevated in IDA mice but normalized in intervention groups (Figure 3C). The spleen is a key organ for red blood cell destruction and iron recycling [18]. The elevated spleen index in the IDA group was a typical manifestation of increased compensatory red cell destruction or extramedullary hematopoiesis under anemic conditions [19]. Normalization of spleen index after beverage intervention indicated that adequate iron supply alleviated the compensatory burden on the spleen. In addition, IDA lowered serum iron and elevated total iron-binding capacity compared to controls (Figure 3D,E). Beverage intervention dose-dependently increased SI and lowered TIBC, demonstrating effective iron absorption and downregulation of compensatory transferrin synthesis. Decreased SI and increased TIBC were core biochemical features of iron-deficiency anemia [20]. Elevated TIBC results from compensatory upregulation of transferrin synthesis to enhance iron transport capacity under iron-deficient conditions. The increase in SI and decrease in TIBC after beverage intervention indicate that exogenous iron was effectively absorbed and utilized to meet bodily demands, thereby downregulating compensatory responses.

These results confirm the successful establishment of the iron-deficiency anemia model, characterized by reduced SI, elevated TIBC, and compensatory increase in spleen index. The dietary polysaccharide-stabilized peptide-Fe^2+^ composite beverage effectively corrected iron metabolism disorders in a dose-dependent manner. None of the interventions induced abnormal body weight changes or heart/liver damage, demonstrating that this composite beverage exhibits good biosafety while effectively supplementing iron.

The present findings were consistent with previous reports. Hu et al. (2024) [21] noted that Antarctic krill heptapeptide–iron complex (LVDDHFL-iron) effectively restored Hb, SI, and TIBC to normal levels. Similarly, Liu et al. (2023) [22] reported that supplementation with oyster protein hydrolysate–iron complex (OPH-Fe) significantly increased SI content and lowered TIBC, with the medium-dose group showing the best effect. These findings collectively supported the potential application value of the dietary polysaccharide-stabilized peptide-Fe^2+^ composite beverage in improving iron metabolism indicators.

### 3.13. Effect of the Composite Beverage on Tissue Iron Content in IDA Mice

The liver serves as the primary storage depot for iron, while the spleen is a key site for iron recycling following erythrocyte destruction [23]. Analyzing the iron content in these two organs can directly reflect the body’s iron storage status and the actual efficacy of iron-supplementation intervention. Hepatic iron content was highest in the control group, reflecting normal iron reserves. In IDA mice, liver iron was significantly reduced due to mobilization to sustain hematopoiesis (Figure 3F). Medium- and high-dose beverage interventions effectively restored hepatic iron to control levels, indicating efficient iron absorption and delivery to liver stores. No difference between medium and high did suggest the medium dose was sufficient for liver repletion. Splenic iron content was also lower in IDA mice (Figure 3G), confirming systemic deficiency. Beverage intervention dose-dependently increased splenic iron. The low dose provided partial recovery, while medium and high doses restored levels to those of the controls. This threshold-dependent response likely reflected prioritization of available iron for hematopoiesis and liver storage before splenic replenishment [24]. In summary, the dietary polysaccharide-stabilized peptide-Fe^2+^ composite beverage dose-dependently reversed the iron-deficient state and effectively restored iron reserves in both the liver and spleen. These findings provided strong evidence at the tissue-storage level for the good bioavailability and iron-supplementing efficacy of this composite beverage.

### 3.14. Effect of the Composite Beverage on Intestinal Oxidative Stress Markers and Inflammatory Cytokines in IDA Mice

**Figure 4:**
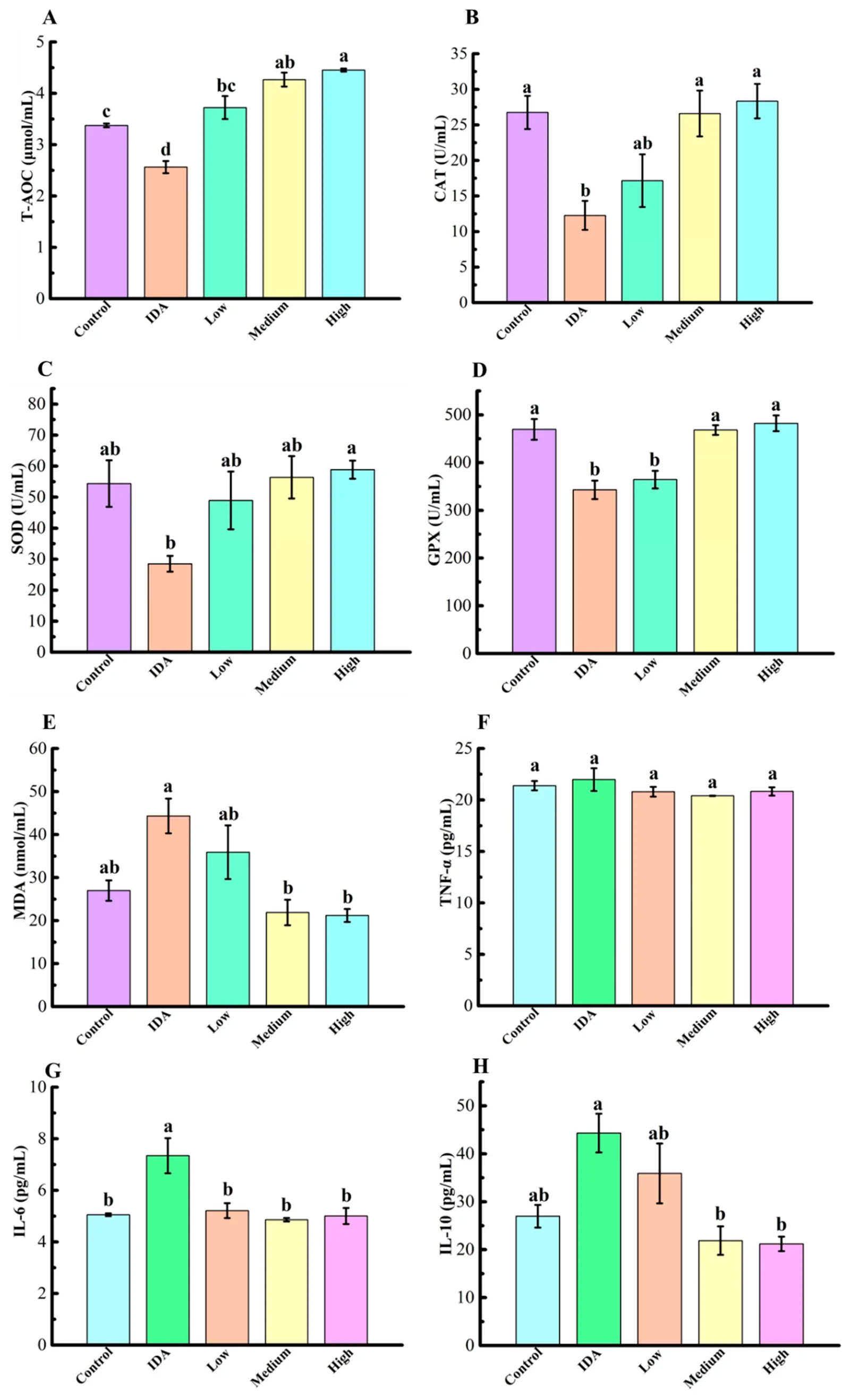
Effects of the composite beverage on duodenal oxidative stress markers (**A**–**E**) and inflammatory cytokines (**F**–**H**) in IDA mice. Different superscript letters above bars indicate significant differences between groups.

To assess intestinal responses to iron supplementation, antioxidant enzyme activities, lipid peroxidation, and inflammatory cytokines were measured in duodenal tissue. Compared with controls, IDA mice showed significantly reduced intestinal T-AOC and activities of CAT, SOD, and GPX (*p* < 0.05) (Figure 4A–D), indicating impaired antioxidant defense. Beverage intervention dose-dependently restored these parameters, with the high-dose group often reaching or exceeding control levels (Figure 4A–D). MDA, a marker of lipid peroxidation, was elevated in IDA mice (*p* < 0.05) but decreased after intervention, especially at medium and high doses (Figure 4E). Furthermore, TNF-α did not differ among groups (Figure 4F). IL-6 was elevated in IDA mice and normalized by all beverage doses (Figure 4G). IL-10 was also higher in IDA mice (*p* < 0.05) (Figure 4H). The elevated IL-10 in IDA mice likely represented a compensatory anti-inflammatory response to mild inflammation associated with iron deficiency. After beverage intervention, the significant decrease in IL-10, coupled with unchanged TNF-α, suggested that the compensatory need for IL-10 was resolved as iron status improved, rather than indicating a treatment-induced pro-inflammatory state. Although IL-10 dynamics in IDA can be context dependent, previous work has shown increased IL 10 expression in the colonic mucosa of anemic animals [25], consistent with our findings. Thus, IDA induced intestinal oxidative stress and altered inflammation [26]. The beverage dose-dependently restored antioxidant defenses, lowered oxidative damage, and normalized inflammatory cytokines without triggering additional stress or inflammation, indicating good intestinal biocompatibility and safety.

These findings align with those reported by Cheng et al. (2021) [25], who also observed decreased CAT, SOD, and GPX activities and increased TBARS and inflammatory cytokine (IL-6, IL-10, TNF-α) levels in colon tissues due to iron deficiency. Supplementation with moderate-dose FeSO_4_ and donkey-hide gelatin peptide–iron complex (EPI) significantly improved these indicators. Moreover, EPI at all doses restored inflammatory cytokine levels to the normal range, further supporting the beneficial role of peptide-Fe^2+^ complexes in alleviating intestinal oxidative stress and inflammatory responses.

### 3.15. Effect of the Composite Beverage on Intestinal Tissue Structure in IDA Mice

**Figure 5:**
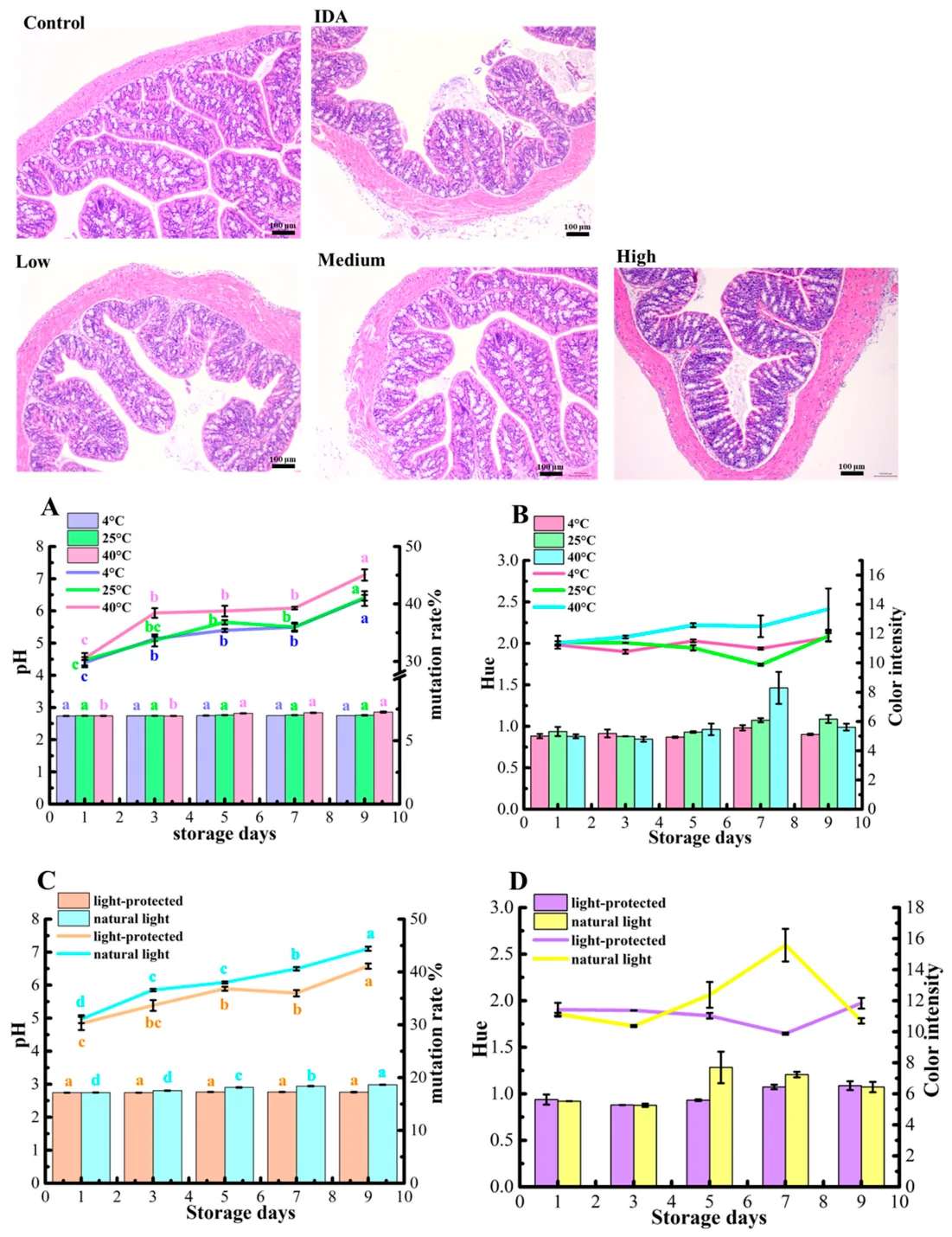
Effects of the composite beverage on colonic tissue in IDA mice and the stability of the beverage under different light and temperature conditions during storage. (**A**) Changes in pH and mutation rate of beverages with prolonged storage time at different storage temperatures. (**B**) Changes in hue and color intensity of beverages with prolonged storage time at different storage temperatures. (**C**) Changes in pH and mutation rate of beverages with prolonged storage time under light-protected/natural light conditions. (**D**) Changes in hue and color intensity of beverages with prolonged storage time under light-protected/natural light conditions. Different superscript letters above bars indicate significant differences between groups.

The control group showed intact colonic mucosa with well-defined glands, abundant goblet cells, normal crypts, and minimal inflammatory infiltration (Figure 5). In contrast, the IDA group exhibited typical mucosal damage, disorganized and atrophic glands, reduced goblet cells, loosely arranged epithelial cells, pale/distorted crypts, and marked inflammatory infiltration, suggesting barrier impairment due to oxidative stress and immune dysregulation. Low-dose intervention partially improved gland atrophy and goblet cell numbers, with reduced but still focal inflammation, indicating limited repair. Medium-dose intervention restored near-normal gland arrangement, significantly increased goblet cells, clarified crypt structure, further reduced inflammation, and normalized mucosal thickness, reflecting effective epithelial and barrier recovery. The high-dose group displayed mucosa histologically similar to controls, with regular glands, abundant goblet cells, clear crypts, and negligible inflammation, demonstrating complete reversal of IDA-induced damage. Thus, iron-deficiency anemia directly damages colonic structure, while the beverage dose-dependently repairs mucosa, likely through improved iron metabolism, antioxidant enhancement, anti-inflammatory effects, and epithelial regeneration.

### 3.16. Effect of Light and Temperature on the Stability of the Composite Beverage During Storage

The rate of change and pH increased during storage at all temperatures (4 °C, 25 °C, 40 °C), with the highest and fastest increase occurring at 40 °C (Figure 5A). This temperature-dependent trend suggested that heat accelerated physical instability, likely by enhancing particle motion and collision, leading to aggregation/sedimentation, whereas 4 °C refrigeration best preserved physical stability [27]. Color intensity decreased and hue shifted over time, with changes most pronounced at higher temperatures (Figure 5B). Fading was likely due to thermal degradation of natural pigments such as anthocyanins, confirming that low-temperature storage was essential for maintaining sensory quality. Light exposure further accelerated increases in the rate of change and pH (Figure 5C), as well as color loss and hue shift (Figure 5D). Natural light, especially UV, can drive photo-oxidation, degrade functional components, and destabilize the colloidal system, while dark storage minimized such photo-catalytic damage. Thus, both elevated temperature and light exposure accelerated beverage deterioration during storage. A “low-temperature, dark-storage” strategy (4 °C, protected from light) was recommended to maximize physical and sensory stability throughout the shelf life.

## 4. Conclusions

This study successfully developed a dietary polysaccharide-stabilized peptide-Fe^2+^ composite beverage with optimized formulation and processing parameters. The beverage effectively addressed limitations of traditional iron supplements, such as low bioavailability and poor stability, through a polysaccharide-based delivery system (FA-COS/PTry-CMC) that enhanced iron stability and absorption. The optimal formulation (cranberry/blueberry concentrate 4:6, 600 mL/L; stabilized complex 200 mL/L; vitamin C 1.5 g/L; pectin 0.06 g/L) and processing conditions (homogenization: 12,000 rpm, 20 min; sterilization: 95 °C, 15 min) ensured high sensory quality and suspension stability. In vivo experiments demonstrated dose-dependent efficacy in reversing IDA in mice, restoring hematological parameters (HGB, SI, TIBC), replenishing hepatic and splenic iron stores, and alleviating intestinal oxidative stress and inflammation. Histopathological analysis confirmed the beverage’s ability to repair colonic mucosal damage. Storage stability was maximized under low-temperature (4 °C) and dark conditions. The beverage outperformed existing peptide–iron complexes in iron delivery efficiency and safety, offering a promising strategy for IDA management. Future work should focus on clinical validation and scalable production.

## Figures and Tables

**Table 1:** Sensory rating reference standards.

Grade	Color (20 Points)	Odor (20 Points)	Taste (30 Points)	Texture (20 Points)	Total Score
Level 1	Deep purple-red, uniform and translucent (18–20)	Rich and harmonious fruit aroma; no metallic or fishy odors (18–20)	Smooth, balanced sweet–sour profile; no off-flavors or aftertaste (28–30)	Clear, uniform liquid; good fluidity; no sedimentation or separation (28–30)	>90
Level 2	Light purple-red, uniform (15–17)	Pronounced fruit aroma; slight metallic or fishy notes (15–17)	Normal sweet–sour balance; slightly bland or viscous; mild aftertaste (25–27)	Uniform with slight sedimentation; no separation (25–27)	80~90
Level 3	Brownish purple-red, non-uniform (10–14)	Weak fruit aroma; noticeable metallic or fishy odor (10–14)	Imbalanced flavor; thin or thick texture; distinct aftertaste (20–24)	Slightly turbid; visible sedimentation (20–24)	60~80
Level 4	Dark brown, dull and patchy (<10)	Unpleasant aroma; strong metallic or fishy odor (<10)	Poor mouthfeel; unbalanced flavor; strong aftertaste (<20)	Turbid; significant sedimentation (<20)	<60

**Table 2:** Orthogonal experiment on basic formula of composite beverage.

	Factors			
Experiment No.	Concentrate Juice	FA-COS@Peptide-Fe^2+^@PTry-CMC	Vitamin C	Blank Column
1	1	1	1	1
2	1	2	3	2
3	1	3	2	3
4	2	1	3	3
5	2	2	2	1
6	2	3	1	2
7	3	1	2	2
8	3	2	1	3
9	3	3	3	1

**Table 3:** Orthogonal experiment on the influence of different factors on the suspension stability of composite beverages.

	Factors			
Experiment No.	Pectin Addition	Homogenization Speed	Homogenization Time	Blank Column
1	1	1	1	1
2	1	2	2	2
3	1	3	3	3
4	2	1	2	3
5	2	2	3	1
6	2	3	1	2
7	3	1	3	2
8	3	2	1	3
9	3	3	2	1

**Table 4:** Results of orthogonal experiment on basic formula of compound beverage.

	Factors				
Experiment No.	Concentrate Juice	FA-COS@Peptide-Fe^2+^@PTry-CMC	Vitamin C	Blank Column	Sensory Score
1	1	1	1	1	89.2
2	1	2	3	2	92.9
3	1	3	2	3	92.3
4	2	1	3	3	92.9
5	2	2	2	1	94
6	2	3	1	2	91.4
7	3	1	2	2	90.8
8	3	2	1	3	88.7
9	3	3	3	1	89.8
K1	91.47	90.97	89.77	91.00	
K2	92.77	91.87	92.37	91.70	
K3	89.77	91.17	91.87	91.30	
R	3.00	0.90	2.60	0.70	
Optimal level	2	2	2	2	

**Table 5:** Results of orthogonal experiment on the influence of different factors on suspension stability of compound beverages.

	Factors				
Experiment No.	Pectin Addition	Homogenization Speed	Homogenization Time	Blank Column	Suspension Stability
1	1	1	1	1	11.74
2	1	2	2	2	8.07
3	1	3	3	3	14.77
4	2	1	2	3	16.63
5	2	2	3	1	10.55
6	2	3	1	2	17.74
7	3	1	3	2	10.97
8	3	2	1	3	8.07
9	3	3	2	1	16.18
K1	11.527	13.113	12.517	12.823	
K2	14.973	8.897	13.627	12.26	
K3	11.74	16.23	12.097	13.157	
R	3.446	7.333	1.53	0.897	
Optimal level	2	3	2	3	

**Table 6:** Effects of different sterilization conditions on microbial indicators of compound beverages.

Sterilization Temperature (°C)	Sterilization Time (min)	Total Plate Count (CFU/mL)
60	5	1.2 × 10^4^
	10	8.5 × 10^3^
	15	3.2 × 10^3^
	20	9.0 × 10^2^
	25	2.1 × 10^2^
70	5	5.0 × 10^3^
	10	1.2 × 10^3^
	15	2.8 × 10^2^
	20	80
	25	60
80	5	3.5 × 10^2^
	10	200
	15	150
	20	45
	25	12
90	5	180
	10	85
	15	15
	20	<10
	25	<10
100	5	<10
	10	<10
	15	<10
	20	<10
	25	<10

## Data Availability

The original contributions presented in this study are included in the article. Further inquiries can be directed to the corresponding authors.
